# Comparative Principles for Next-Generation Neuroscience

**DOI:** 10.3389/fnbeh.2019.00012

**Published:** 2019-02-05

**Authors:** Cory T. Miller, Melina E. Hale, Hideyuki Okano, Shigeo Okabe, Partha Mitra

**Affiliations:** ^1^Cortical Systems and Behavior Laboratory, Neurosciences Graduate Program, University of California, San Diego, San Diego, CA, United States; ^2^Department of Organismal Biology and Anatomy, University of Chicago, Chicago, IL, United States; ^3^Department of Physiology, Keio University School of Medicine, Tokyo, Japan; ^4^Laboratory for Marmoset Neural Architecture, RIKEN Center for Brain Science (CBS), Wako, Japan; ^5^Department of Cellular Neurobiology, Graduate School of Medicine and Faculty of Medicine, University of Tokyo, Tokyo, Japan; ^6^Cold Spring Harbor Laboratory, Cold Spring Harbor, NY, United States

**Keywords:** phylogeny, neuroscience, molecular-genetics, evolution, homology (comparative) modeling, brain evolution

## Abstract

Neuroscience is enjoying a renaissance of discovery due in large part to the implementation of next-generation molecular technologies. The advent of genetically encoded tools has complemented existing methods and provided researchers the opportunity to examine the nervous system with unprecedented precision and to reveal facets of neural function at multiple scales. The weight of these discoveries, however, has been technique-driven from a small number of species amenable to the most advanced gene-editing technologies. To deepen interpretation and build on these breakthroughs, an understanding of nervous system evolution and diversity are critical. Evolutionary change integrates advantageous variants of features into lineages, but is also constrained by pre-existing organization and function. Ultimately, each species’ neural architecture comprises both properties that are species-specific and those that are retained and shared. Understanding the evolutionary history of a nervous system provides interpretive power when examining relationships between brain structure and function. The exceptional diversity of nervous systems and their unique or unusual features can also be leveraged to advance research by providing opportunities to ask new questions and interpret findings that are not accessible in individual species. As new genetic and molecular technologies are added to the experimental toolkits utilized in diverse taxa, the field is at a key juncture to revisit the significance of evolutionary and comparative approaches for next-generation neuroscience as a foundational framework for understanding fundamental principles of neural function.

## Introduction

**Figure 1:**
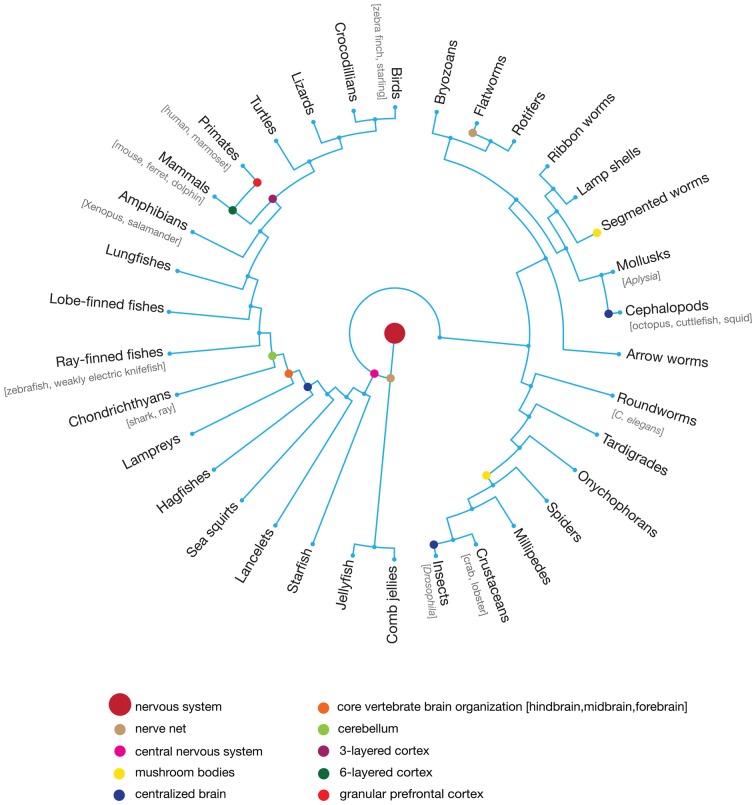
Circular evolutionary tree of representative animal taxa that emerged following the evolution of the nervous and vestibular systems. While junctions of branches represent the degree of phylogenetic relatedness over evolution, distance along the tree does no scale with actual time in natural history. Colored circles indicate last common ancestor for phylogenetic groups that exhibited a particular characteristic of the nervous system which can be used to reconstruct shared, unique and convergent features of nervous systems. The central nervous system emerged early in animal evolution and is homologous across multiple taxa, while granular prefrontal cortex is a unique property of primate brains. Likewise, the independent evolution of centralized brains in vertebrates and multiple invertebrate taxa—insects and cephalopods—represents an example of convergent evolution. The more commonly used “model” organisms in neuroscience are listed in brackets below their taxonomic groups, though this list is not exhaustive.

There are over 1.5 million described, living species of animals; all but a few thousand have nervous systems and nervous system-generated behaviors. Like all characteristics of organisms, nervous systems and behaviors evolve by descent with modification in which selective forces can preserve ancestral traits and amplify freshly generated variation (Figure 1). Selection on nervous system anatomy occurs indirectly, as an intermediate to the genome, where variation originates. It is function—including behavior—rather than structure that is under direct evolutionary selection. Conversely, behaviors are constrained by nervous system architecture, which in turn is determined by a developmental program encoded in genomes (Alexander, 1974; Emlen and Oring, 1977; Agrawal, 2001; Lamichhaney et al., 2015; Session et al., 2016) and phylogenetic history (Ryan et al., 1990; Shaw, 1995; Rosenthal and Evans, 1998; Ng et al., 2014; Odom et al., 2014). Yet despite such constraints imposed on how these systems evolve, plasticity afforded by various processes throughout the nervous system affect how behaviors actually manifest in individuals in response to its unique experiences in the environment (Meyrand et al., 1994; Gross et al., 2010). Ultimately these dynamic relationships have undoubtedly fueled many facets of biological diversity. While the modern experimental toolkit has provided unprecedented glimpses into the intricacies of neural systems, phylogenetic approaches that leverage species differences are pivotal keystones for elucidating the structure and function of neural architecture.

The arrival of genetically encoded tools to investigate neuronal circuitry has accelerated our rate of discovery in the past decades, but it has come at a cost to the study of species diversity. Ironically, comparative neuroscience that explores a range of species, nervous system organizations, and behaviors has convincingly shown that detailing the nuances of neuronal microcircuitry are critical to understanding behavior (Kepecs and Fishell, 2014; Ben Haim and Rowitch, 2017; Real et al., 2017; Wamsley and Fishell, 2017). However, with an increased reliance on a small number of animal models, we are often left making assumptions about whether a discovered neuronal process reflects a common principle of brain organization and function or is specific to a particular taxon and its biology. Without well-framed, phylogenetically informed species comparisons, the significance of differences between any two species is difficult to understand. Both rodents and primates, for example, have afferent dopaminergic projections from the substantia nigra pars compacta (SNc) to the striatum (nigrostriatal pathways). But whereas in primates the SNc also project to areas of the dorsolateral frontal cortex (nigrocortical pathways), the analogous pathways are essentially absent in rodents (Wiliams and Goldman-Rakic, 1998). Interestingly, the emergence of nigrocortical pathways in primates is correlated with a marked increase of dopamine receptors in frontal cortex (Murray et al., 1994; Düzel et al., 2009). Determining the functional significance of this circuit difference on behavior cannot be ascertained without a more thorough understanding of differences across mammalian taxa more closely related to primates—tree shrews (Scandentia) and flying fox (Dermoptera)—and closer relatives of rodents, such as rabbits (Lagomorpha). Ultimately, these species’ nervous systems comprise some characteristics that are homologous due to common ancestry, some characteristics evolved due to shared selection pressures—reflecting convergence across taxa—and other characteristics that are unique (Figure 1; Kaas, 2013; Karten, 2013; Roth, 2015). Both shared and uniquely adapted characteristics have illuminated our understanding of nervous systems, often in different and complementary ways, but distinguishing between these possibilities can only be accomplished through comparative research. Leveraging the extraordinary resolution afforded by modern molecular technologies within a comparative framework offers a formidable approach to explicating the functional motifs of nervous systems.

The single most powerful method for identifying common principles of neural circuit organization is phylogenetic mapping. Characteristics are mapped onto a well-supported phylogenetic tree that is of appropriate resolution and species richness to the question being addressed (Felsenstein, 1985; Harvey and Krebs, 1991; Harvey and Pagel, 1991; Clark et al., 2001; Krubitzer and Kaas, 2005; Hale, 2014; Striedter et al., 2014; Liebeskind et al., 2016). This approach makes it possible to distinguish the evolutionary origin of a particular property of the brain or nervous system and generate testable hypotheses about its functional significance based on phylogenetic history (Barton et al., 2003; Harrison and Montgomery, 2017; Laubach et al., 2018). In some cases, homologous traits have a long history—such as the hindbrain or spinal column of vertebrates (Hirasawa and Kuratani, 2015)—whereas others only occur in small groups of closely related organisms or single species (Gould, 1976; Catania and Kaas, 1996; Douglas et al., 1998; Shepherd, 2010; Albertin et al., 2015). Characteristics that appear in multiple groups independently are examples of convergent evolution, or homoplasy. Notably, both homologous and homoplasious features have illuminated our understanding of nervous systems. Discoveries of common principles reveal the core building blocks of nervous systems or architectural features that may have biomimetic utility in engineering. Likewise, specialist adaptations in highly niche-adapted species yield critical data on how specific neural circuits evolved to solve key challenges and can serve as powerful heuristics for investigating other species, including humans. Each scenario for nervous system evolution offers the opportunity to better understand neural function and elucidate their dynamical processes. A phylogenetic framework offers a valuable tool to next-generation neuroscience that, when wielded correctly, can drive new frontiers of discovery not only in classic biological disciplines but in fields involving human-engineered systems, such as artificial intelligence and robotics.

Recent studies demonstrate the power of phylogenetic tools in addressing critical questions in neuroscience (Montgomery et al., 2011; Laubach et al., 2018). Gómez-Robles et al. (2017), for example, used evolutionary simulations and a multiple-variance Brownian motion framework to reconstruct hypotheses of ancestral states to examine the classic hypothesis of a relationship of dental reduction to brain size increase in hominins. Their data rejected this idea and indicated different patterns of evolution for tooth reduction and for brain size. Likewise, DeCasien et al. (2017) tested the social brain hypothesis that the large size and complexity of the human brain were driven by increasing social complexity and advantage of a larger, more complex brains in primate ancestors. The researchers used phylogenetic generalized least squares regression of traits with a rigorously derived phylogeny based on the 10KTrees primate resource and other controls. This rigorous analysis showed that sociality is better predicted by diet, specifically frugivory, than it can be explained by other social factors. They suggest that various aspects of foraging, such as retaining complex spatial information, may have benefitted from a larger and more complex brain. These examples illustrate how a phylogenetic framework offers a valuable tool to modern neuroscience, but new approaches make it possible to ask even more precise questions.

Advances in modern molecular neuroscience make it possible to further refine comparative questions about nervous systems by more explicating the relationship between genes and phenotypic expression. This can be accomplished by measuring the strength of evolutionary selection on a gene by calculating a dN/dS ratio (e.g., selection+neutral/neutral). In this approach, a ratio below 1 would indicate negative selection acting on the gene, whereas positive selection would be indicated by a ratio greater than 1. By comparing dN/dS ratios across a large number of species, one can more precisely map positive and negative evolutionary changes within the nervous system (Enard, 2014). For example, primates have notably high encephalization quotients (i.e., brain to body size ratio) than other mammals but questions remain about the evolutionary forces that drove this facet of selection in primates (Preuss, 2007; Dunbar and Shultz, 2017), including the genes that regulated the change. Notably not all primates have large brains, as the overall size of species’ brains within this order varies considerably ranging from large bodied apes on one end of the spectrum, such as humans and gorillas, and Callitrichid monkeys—tamarins and marmosets—on the other end. This latter family of New World monkeys has notably undergone miniaturization during primate evolution (Harris et al., 2014; Miller et al., 2016). To more precisely explore questions of brain size evolution within primates, Mongtomery and colleagues calculated dN/dS ratios for several genes associated with microcephaly across 20 anthropoid monkey species (Montgomery et al., 2011; Montgomery and Mundy, 2012b). While at least three genes revealed positive selection across these primate species, the most compelling case for a genetic correlate of brain size in primates was for *ASPM*. This gene not only covaried with increased brain size across most primates, but a decrease in brain size in the small bodied Callitrichid monkeys (Montgomery and Mundy, 2012a). While brain size is one broad phenotype in which to perform such comparative analyses, the same phylogenetic approach could be utilized across numerous properties of a nervous systems’ functional architecture and behavior (Krubitzer and Kaas, 2005). When wielded correctly, this powerful comparative method can be implemented to resolve existing debates and drive new frontiers of discovery.

## Comparative Neurobiology in the 21st Century

The utilization of phylogenetics in neuroscience has a long and rich history. In mammals, detailed neuroanatomical investigations complementing quantitative studies of behavior across a diversity of species have fueled hypotheses about the functional organization of nervous systems and the mechanisms underlying a diversity of neural processes (Wells, 1978; Young, 1978; Kaas, 1986, 2013; Karten, 1991; Strausfeld, 1995, 2009, 2012; Kotrschal et al., 1998; Strausfeld et al., 1998; Krubitzer, 2000; Rodríguez et al., 2002; Jarvis et al., 2005; Krubitzer and Kaas, 2005; Krubitzer and Seelke, 2012; Striedter et al., 2014). In many respects, the limiting factor to explicating these hypotheses has been the available functional tools to examine the mammalian brain *in vivo* with the same level of detail available to neuroanatomists. Despite its precise temporal and spatial resolution, neurophysiological recordings are largely blind to the finer details of neural architecture—such as cell types and layers—while the poor spatial and temporal resolution of functional neuroimaging limits its utility to examine key cellular and population-level processes fundamental to nervous system function. Likewise, traditional techniques to functionally manipulate the neural structure, such as electrical microstimulation and pharmacological manipulations, generally impact relatively large populations of neurons. Due to such technological limitations, experimental questions have historically been constrained to broader-scale issues about brain function, such as the role of particular areas or nuclei for a given behavior or task. The development of next-generation molecular technologies opened the door to examine nervous systems with a level of resolution that was not previously possible (Stosiek et al., 2003; Boyden et al., 2005; Mank et al., 2008; Deisseroth, 2015). Perhaps not surprisingly, many of the functional data emerging from the implementation of these molecular methods have supported established anatomical observations and conceptual models suggesting that the fine details of neural circuitry—cell types, patterns of projections, connectivity, et cetera—are pivotal to describing the functional architecture of neural systems that support behavior. Leveraging the power of precision afforded by genetic tool kits in order to explore functional circuitry is a defining feature of modern neuroscience, yet a continued appreciation of species diversity within a phylogenetic framework may be essential to unlock the deepest mysteries of nervous systems (Carlson, 2012; Yartsev, 2017).

A key advantage of a comparative framework in neuroscience is that it provides a powerful tool for testing hypotheses of structure and function in nervous systems. The significance of establishing homology and examining convergent systems is highlighted by work in the motor system of sea slugs where phylogenetically framed studies have shown that what might be classified as a single behavior in this group of organisms has arisen multiple times and with different neural circuit underpinnings (Katz, 2011, 2016). Multiple evolutionary events leading to an association of traits can also support arguments for the relationship between structure and function that might be predicted but not testable by studying one or several individual species without consideration of phylogeny. For example, Aiello et al. (2017) argued an example of the evolutionary tuning of mechanosensation to biomechanical properties of fish fins. They showed that while the basal condition was a very flexible fin consistently across multiple lineages, when stiff fins evolved there was corresponding increase in the sensitivity of mechanosensory afferent (Aiello et al., 2017). In both sea slug and fish examples, access to a group of closely related organisms with a known phylogeny was essential. Such comparative phylogenetic framing would be of limited value among the few traditional genetic model organisms. Ultimately, most species’ neural systems comprise each of these characteristics, reflecting common principles that were inherited and maintained and the evolution of derived mechanisms to support idiosyncratic behaviors of the species. A comparative framework not only allows one to make these distinctions but to determine whether a characteristic is itself an adaptation or the byproduct of other evolutionary forces—a spandrel (Gould and Lewontin, 1979)—with little functional significance.

Consider, for example, the mammalian neocortex. This six-layered brain structure is unique to the taxonomic group and its occurrence in all extant mammals suggesting that it evolved early in mammalian evolution when these synapsids first emerged ~300 mya (Krubitzer and Kaas, 2005). Comparative anatomy and physiology suggest that many characteristics of the avian and reptilian brain—comprised of nuclei and a three-layered cortex—are shared with the mammalian brain (Jarvis et al., 2005; Karten, 2013; Calabrese and Woolley, 2015). Neurons and circuits do not arise *de novo* as new or altered functions evolve, but rather are adapted from preexisting morphology and developmental programs. The evolutionary history of neurons and circuits and how they differ among taxa provide critical information for interpreting circuit organization in related taxa. Dugas-Ford et al. (2012) used fluorescence *in situ* hybridization to examine expression of genes to show that cell types within the mammalian neocortical layer IV input and layer V output circuit are homologous with the parallel substrates in the avian brain. Consistent with various evolutionary examples, this suggests that many of the computational foundations of the sauropsid brain were conserved during the evolution of neocortex, presumably because they remained optimal for facets of neural function (Shepherd and Rowe, 2017). We must, however, also ask what computational advantage the derivation of the 6-layered neocortex may have afforded mammals that were constrained by the functional architecture of the avian/reptilian brain (Shepherd, 2011), particularly given the metabolic costs associated with the increased encephalization quotient in neocortex (Isler and van Schaik, 2006). A strategy involving detailed behavioral and neuroanatomical comparisons across species implemented in tandem with modern molecular technologies is ultimately needed to resolve these issues.

## The Next Frontier

The statistician George E. P. Box famously stated that “All models are wrong, but some are useful” (Box, 1979). Revisiting this sentiment is particularly meaningful at this point in time because of our increased reliance on “model” organisms in neuroscience today (Brenowitz and Zakon, 2015; Goldstein and King, 2016; Yartsev, 2017). Whereas anatomical data have historically come from an impressive diversity of species, the weight of work implementing modern molecular approaches in nervous systems has been performed on increasingly fewer animal species. In most cases, these species have been selected for study due to their amenability to transgenic manipulation of their genome, but without clear understanding of the evolutionary origins of the traits being investigated. In some model organisms, for example, the ease of culture and embryo manipulation, limited neuron population size, and accessibility into the nervous system have provided opportunities to investigate neurons and circuits at levels not possible in humans (e.g., *C. elegans*, White et al., 1986; Venkatachalam et al., 2015; Markert et al., 2016; Jang et al., 2017; Yan et al., 2017), fruit fly (Malsak et al., 2013; Nern et al., 2015; Fushiki et al., 2016), zebrafish (Liu and Fetcho, 1999; Ahrens et al., 2012, 2013; Nauman et al., 2016; Hildebrand et al., 2017), and mice (Glickfeld et al., 2013; Issa et al., 2014; Glickfeld and Olsen, 2017; Guo et al., 2017). By focusing inquiry to these genetic models, we have made considerable discoveries about particular facets of these neural systems. At the same time, the limits of this strategy are increasingly evident. To assume that any single species represents an archetypal brain with unquestioned parallels to humans belies a misunderstanding of evolutionary forces that drive the phylogenetic diversity of nervous systems, particularly given the many known neuroanatomical, physiological and genetic differences across taxa (Bolker, 2012). Superficial similarities may mislead, as brains ultimately should be examined and data interpreted in the context of a species taxonomic lineage. While broad species comparisons can identify gross level similarities, the tactic of leveraging molecular technologies to more precisely explicate shared and derived characteristics of nervous systems across diverse taxa has the potential to be the spine in the next chapters of Neuroscience.

The challenges of utilizing a single model organism—mice—as a model of human disease from a phylogenetic perspective is clearly evident in the context of neuropharmacology. Neuropharmaceuticals identified in mouse-model screens have notoriously failed human clinical trials (Hyman, 2013). Despite the importance of this issue, few failed clinical trials have been investigated retrospectively and the underlying problems remain. This situation necessitates new technologies and phylogenetic approaches to address this fundamental gap. In particular, a revolutionary new technology named Drugs Acutely Restricted by Tethering (DART) offers an unprecedented capacity to selectively deliver clinical drugs to genetically defined cell-types, offering a means to revolutionize our understanding of the circuit mechanisms of neuropharmacological treatments (Shields et al., 2017). Comparative biology will be critical in realizing the full potential of such novel methodologies (Goldstein and King, 2016). For example, while the findings offered by DART in a mouse model of Parkinson’s disease were remarkable, it remains to be tested whether similar effects would occur in primates, including humans, given substantive differences in the basal ganglia between rodents and primates (Petryszyn et al., 2014). These cross-taxa differences include the division of the dorsal striatum into two distinct structures—caudate nucleus and putamen—in primates (Joel and Weiner, 2000). The known circuit differences likely reflect important properties of how the areas of basal ganglia interact with neocortex to support aspects of primate motor behavior that are distinct from those in rodents. It is differences in both functional brain architecture and broader physiology that limit the predictive value of mice as a model of human disease (O’Collins et al., 2006; Sena et al., 2006; Manger et al., 2008; Lin et al., 2014; Grow et al., 2016; Perlman, 2016). By implementing molecular tools within a phylogenetic framework, the functional differences between species could be more precisely examined—at multiple scales of molecular and cellular specificity—to more explicitly test their relationship, identify the key sources of variance and, therefore, increase translational success.

Beyond biomedical implications, a comparison of neural network architecture across taxa in the context of selective behaviors may help design artificial neural networks tailored to artificial intelligence tasks that were previously intractable. Even the simple ideas—such as reinforcement learning—when implemented suitably in the modern context, has yielded automated programs that can defeat humans at the game of Go (Silver et al., 2017). Such a task was previously deemed too difficult for computational approaches. However, the theoretical basis of the improved performance of these artificial neural networks is only beginning to be understood (Marcus, 2018). The comparative approach—and its potential for leading to theoretical understanding—promises to be important for engineering and social applications outside of biomedicine.

Describing the full, synapse by synapse, connectivity of a neural network, dubbed its “connectome,” provides unmatched structural information to inform organizational principles and function and to interpret associated network physiology and behavior. Here use of biodiversity and phylogenetically-informed taxon selection and comparisons would provide exceptional value. Complete reconstructions of processes in the neuropil and synaptic connectivity matrices are being obtained in the nervous systems of invertebrates including the foundational full network model of *C. elegans* (White et al., 1986), as well as *Drosophila* (Takemura et al., 2017a,b), *Platynereis* (Randel et al., 2014), and *Hydra* (Bosch et al., 2017). These species have significantly smaller nervous systems and fewer neurons and thus are more tractable than vertebrates for comprehensive circuit analysis. One drawback of the electron microscopy (EM) based reconstructions is the lack of information about neurotransmitters and neuromodulators. However, correlative physiological information is now possible to obtain by measuring activity using Ca^++^ indicators (Bock et al., 2011). This indicates the need to combine data sets across modalities. As yet, the distribution of such EM reconstructions across the phylogeny is sparse. As these data sets grow and the number of species studied broadens, there will be increased opportunities to compare across taxa. For such comparisons of networks a phylogenetic framework will be critical for interpreting variation across taxa (Katz, 2011; Katz and Hale, 2017).

A diverse set of species have laid the foundation for our field (Figure 1). Although we have increasingly relied on a handful of genetic models to push new frontiers of discovery, the stage is set to expand that empirical horizon considerably. As the process of developing new genetically modified organisms becomes easier and cheaper (Sparrow et al., 2000; Sasaki et al., 2009; Takagi et al., 2013; Abe et al., 2015; Okano et al., 2016; Park et al., 2016; Sato et al., 2016; Okano and Kishi, 2018), the potential for the CRISPR/Cas9 system to be applied across many taxa (Niu et al., 2014; Tu et al., 2015) and the increased selectivity afforded by viral approaches (Dimidschtein et al., 2016), the feasibility of applying powerful next-generation molecular tools to a broader diversity of species is increasingly possible (Leclerc et al., 2000; Izpisua Belmonte et al., 2015; Sadakane et al., 2015; Ferenczi et al., 2016; Liberti et al., 2016; MacDougall et al., 2016; Picardo et al., 2016; Roy et al., 2016; Santisakultarm et al., 2016; Kornfeld et al., 2017; Shields et al., 2017). Furthermore, other non-genetic technological advances, such as those involved in systematic mapping of neural architecture at EM and light microscopy (LM) scales (Bohland et al., 2009; Osten and Margrie, 2013; Oh et al., 2014; Kornfeld et al., 2017), as well as associated advanced analytical methods (Helmstaedter and Mitra, 2012), will likely generalize more easily across taxa and offer powerful complementary approaches. With a rapidly expanding toolkit comprised of more traditional and modern techniques available to probe different nervous systems, incredible biological diversity available that has yet to be explored, and phylogenetic tools to interpret neural characteristics within a comparative framework, the coming years are set to be a particularly exciting time to forge new frontiers in our field.

## Author Contributions

CM and MH were the lead authors on this article, but all contributed significant feedback at various stages of writing.

## Conflict of Interest Statement

The authors declare that the research was conducted in the absence of any commercial or financial relationships that could be construed as a potential conflict of interest.
